# Where next for understanding race/ethnic inequalities in severe mental illness? Structural, interpersonal and institutional racism

**DOI:** 10.1111/1467-9566.13001

**Published:** 2019-09-27

**Authors:** James Y. Nazroo, Kamaldeep S. Bhui, James Rhodes

**Affiliations:** ^1^ Cathie Marsh Institute University of Manchester Manchester UK; ^2^ Queen Mary University of London London UK; ^3^ Department of Sociology University of Manchester UK

**Keywords:** racism, psychosis, disparity, inequality, ethnicity, race

## Abstract

In this article we use the example of race/ethnic inequalities in severe mental illness to demonstrate the utility of a novel integrative approach to theorising the role of racism in generating inequality. Ethnic minority people in the UK are at much greater risk than White British people of being diagnosed with a severe – psychosis related – mental illness, and this is particularly the case for those with Black Caribbean or Black African origins. There is entrenched dispute about how we might understand the drivers of this inequality. To address this dispute we build on, and to a certain extent refine, established approaches to theorising structural and institutional racism, and integrate this within a theoretical framework that also incorporates racist/discriminatory interactions (interpersonal racism). We argue that this provides a conceptually robust and thorough analysis of the role of inter‐related dimensions of racism in shaping risks of severe mental illness, access to care, and policy and practice responses. This analysis carries implications for a broader, but integrated, understanding of the fundamental drives of race/ethnic inequalities in health and for an anti‐racism public health agenda.

## Introduction

Ethnic minority people are more likely to be diagnosed as having a severe – psychosis related – mental illness than the white majority in the UK and this is particularly the case for those with Black Caribbean or Black African origins. A recent meta‐analysis gave a pooled risk ratio of incident diagnosed schizophrenia in the Black Caribbean population of 5.1 (95% CI 4.2–6.2), an even higher risk for Black African people (6.0, 95% CI 3.5–10.2) and a raised risk for South Asian and White minority groups (respectively 2.3, 95% CI 1.6–3.3 and 2.5, 95% CI 1.7–3.6) (Halvorsrud *et al*. 2019). Similar findings have been reported in other developed countries (Bresnahan *et al*. 2007, Cantor‐Graae *et al*. 2005, Selten *et al*. 2001, Veling *et al*. 2006). Importantly, the UK findings have been reported consistently for more than 60 years and appear to be persistent over time and across generations (Bagley 1971, Cochrane and Bal 1989, Halvorsrud *et al*. 2019, Harrison *et al*. 1988, Kiev 1965, King *et al*. 1994, McGovern and Cope 1987, Pinsent 1963, Van Os *et al*. 1996), despite numerous policy interventions such as the Inside Outside report (Sashidharan 2003), the Delivering Race Equality Action Plan (Department of Health 2005) and recommendations from the Joint Commissioning Panel for Mental Health (2014). The findings include also an even greater and persistent over‐representation at the severe end of the diagnostic spectrum, as reflected in rates of compulsory treatment and contact with the criminal justice system prior to treatment (Bhui *et al*. 2015, Halvorsrud *et al*. 2018, Morgan *et al*. 2005), despite no consistent evidence of greater violence or substance misuse (Bhui *et al*. 2015).

Somewhat surprisingly, this level of excess risk is not fully reflected in population surveys, where the prevalence of symptoms of severe mental illness, and consequent estimate of risk of psychotic illness, among Black Caribbean people is of the order of two to three times higher than that for the White majority population (Nazroo 1997, Nazroo and King 2002, Qassem *et al*. 2015) – a still very high rate in comparison with ethnic differences in other health outcomes, but considerably lower than the rates of diagnosis and compulsory treatment.

Although these high rates have been repeatedly documented (Halvorsrud *et al*. 2018) and are often related to social and economic disadvantage, there is entrenched dispute about how we might understand the drivers of these inequalities (Fernando 2004, Singh and Burns 2006). Typically there has been a focus on individual risk, generated as a result of such things as socioeconomic deprivation, discrimination and exposure to childhood trauma. Such approaches, however, pay insufficient attention to the ways in which these risk factors, and institutional responses to them, are shaped by processes related to racism. Indeed, claims around the role of racism and how racism shapes the provision of mental health services have divided the field, with some claiming that institutional racism is core to the operation of contemporary mental health services (Fernando 2004), and others arguing that there is no evidence for a role for racism *per se*, and that the focus on racism is both misleading and harmful, because it runs the risk of denying people appropriate care (Singh and Burns 2006).

Core to this dispute is the lack of a conceptually robust and thorough analysis of the role of various inter‐related dimensions of racism in shaping risks of severe mental illness, access to care, and policy and practice responses. To address this lack, here we first discuss approaches to understanding the relationship between ethnicity and health. Second, we describe a novel approach to theorising inter‐related dimensions of racism – we heuristically classify racism along the dimensions of structural, interpersonal and institutional, which empirically overlap and are interdependent. Third, we examine the evidence for the relationship between interpersonal and structural racism and race/ethnic inequalities in the risk of severe mental illness. And, fourth, we examine evidence for the operation of racism within mental health institutions. We conclude by arguing that the value of this novel approach is that it allows for an integration of both an understanding of individual risk of illness, together with the health system response, and places this within a broader structural frame to understand how and why these processes are patterned beyond the individual. We also argue that the novel theoretical approach to understanding the role of racism that is developed in this article has a broader utility in understanding ethnic/race inequalities in health and other outcomes.

## Approaches to understanding race/ethnic inequalities in health

Within the epidemiological and clinical literature, race/ethnic groups are typically treated as unproblematically real, pre‐constituted, entities representing embodied difference, without attention paid to the historical and contemporary contexts within which they are constructed and given meaning. This approach allows for an examination of differences in disease risk across ethnic groups, with the hope of understanding more about the aetiology of specific diseases by examining the characteristics of those race/ethnic groups most at risk (Senior and Bhopal 1994). The challenge for such research then becomes empirical, how to accurately assess race/ethnic identity (Bhopal 2014), with the nature of what constitutes race/ethnicity often left untheorised (Nazroo 1998). Rather, race/ethnic groups are considered to have pre‐specified properties, be they genetic, cultural or relational (where ‘otherness’ is somehow seen to reflect natural differences), that become the source of explanation for inequalities in outcomes. So, even where the focus is on the socioeconomic disadvantage associated with race/ethnicity, attention is very rarely paid to the processes that lead to this disadvantage, how it is shaped by the reification and devaluation of race/ethnic identities, and how this then shapes life chances, interpersonal interactions and encounters with institutions.

Our argument is not to deny that race/ethnicity has real effects, rather that these effects are a result of the historically and politically shaped meanings ascribed to race/ethnic identities, a process that we and others describe as racialisation (Hughey and Jackson 2017). Indeed, Emirbayer and Desmond (2015) have argued that we need to consider how race/ethnic groups are configured in relational terms within social spaces and how this reflects structural, cultural and symbolic negotiations of power. Central to these processes are economic, cultural, legal, political and symbolic resources that shape how identities are perceived, valued, mobilised and interacted with. This, then, has direct impacts on both risk of illness – in terms of material and psychosocial stresses (including the risk of internalised racism, Jones 2000) – and interactions with institutions. Of specific importance here is to consider how emotions attached to symbolic resources, in particular shared emotions around risk, danger, fear and disgust, shape the practices of individuals and institutions.

Following this, we argue that to achieve an adequate understanding of race/ethnic inequalities in health we need to understand the ways in which identities are racialised and the consequent substantial impacts on the lives of ethnic minority people. To do this, we first consider the ways in which structural racism leads to disadvantage in accessing key economic, physical and social resources. Then, we examine how interpersonal racism, from everyday slights, through discrimination in a range of settings, to verbal and physical aggression, emphasises the devalued and risky status of both those who are directly targeted and those who have similarly racialised identities, thereby engendering meaningful psychosocial stress. Finally, we place a particular emphasis on the role of institutional racism. In doing so, we move beyond common definitions of institutional racism, which focus on discrimination flowing from processes and procedures, rather than intention, and go on to examine how the systems of operation in institutions relate to both structural and interpersonal racism, which are reflected in routine procedures, in cultures of practice and the collective‐emotional regulation of relationships. All of these then translate into actions that shape the experience of racialised groups within these institutions. We detail our approach to understanding these three forms of racism in the next section. While acknowledging that our approach to this classification is a heuristic device (see Hicken *et al*. 2018, Hughey and Jackson 2017 for alternative approaches), we argue that this allows for a novel, integrative, theoretical approach that allows us to draw on and develop existing approaches to understanding racism in order to provide a fundamental explanation for race/ethnic inequalities in health, and one that could also be usefully applied to other, non‐health, outcomes.

## Conceptualising racism

Racisms do not exist in singular or static forms. As historically and politically determined systems of domination, racisms work to exclude, marginalise and inferiorise groups on the basis of purported physical, cultural and symbolic differences (Golash‐Boza 2016, Goldberg 1993). Racisms operate as part of a wider system of racialisation in which racial/ethnic collectivities are constituted and given meaning, status and value within particular ‘racial orders’ (Emirbayer and Desmond 2015). According to Omi and Winant (1994), modern societies are thoroughly imbricated with race thinking – representing ‘racial formations’ or ‘racial states’ – within which ideas of, and categorisation by, race are central to their organisation and regulation. While an invocation of race/ethnicity is not necessarily a manifestation of racism, even though it reflects racialised thinking, such an invocation is necessary to provide a means through which racialised inequalities are enacted and enshrined. Song (2014) has suggested that we should view racism as a particular form of racialisation. Drawing on Omi and Winant (1994), she argues that racism is present not simply wherever ‘race’ is invoked or acted upon, but where ‘it creates or reproduces structures of domination based on essentialist categories of race’ (Song 2014). Similarly, Golash‐Boza (2016) argues that racism comprises an ideology where physical difference is linked to cultural and social difference, thereby allowing populations to be placed on a hierarchal scale and allowing for the subordination of those considered to be inferior.

If racism is about the institution and reproduction of race/ethnic disadvantage, a central challenge for contemporary studies of racism is to move beyond simply establishing the existence and/or extent of racism and race/ethnic inequalities to instead ‘better understand the structures and processes of racial inequality’ (Emirbayer and Desmond 2015). Here there is a need to more fully integrate the macro, meso and micro dimensions of racism and racialised inequality and the ways in which they interact (Emirbayer and Desmond 2015, Phillips 2010, Song 2014). This leads us to our heuristic classification of structural, institutional and interpersonal racism. While much theoretical and empirical work investigating race and ethnicity and racialised inequalities – including in the area of mental health – has focused on specific domains (particularly institutional racism), there has been a tendency to neglect the inter‐relations between these scales of racism. Adopting a wider, more integrated, approach this article offers instead a frame through which racial and ethnic inequalities can be more adequately located, explicated and accounted for.

Any consideration of racism must necessarily focus on the structural, macro, level. Operating alongside and in interaction with other axes of domination, such as class and gender (Byrne 2015, Golash‐Boza 2016, Phillips 2010, Song 2014), race/ethnicity remains a key determinant of social location, status and power. The dominance of macro‐sociological accounts of race/ethnic inequalities reflects the ways in which racism is seen to inhere in the very fabric of contemporary societies (Omi and Winant 1994). Here, the legacies of historical regimes of colonialism, race‐based slavery and apartheid interact with current processes of globalisation, migration and governance, continuing to shape present day inequalities in accessing key economic, physical and social resources (Bailey *et al*. 2017, Phillips 2010). Importantly, structural racism consists of not just material, but also cultural and ideological dimensions (Essed 1991). The circulation of ideas and representations that produce race and ethnic groups as different, but also as threatening and inferior, serve to rationalise and inform an uneven distribution of resources. They comprise the co‐constitution of material with symbolic denigration. As Bonilla‐Silva (1997) argues, ‘racialized social systems’ work to distribute ‘economic, political, social, and even psychological rewards to groups along racial lines’. Here our approach differs somewhat from that adopted by Hicken *et al*. (2018), who instead argue for a distinction between the structural and cultural domains. Instead, as described, we see the cultural as embedded within, and a crucial dimension, of the structural, even though it is played out in both institutional practices and interpersonal interactions.

Closely related to these cultural and ideological domains, Emirbayer and Desmond (2015) identify the significance of the ‘collective‐emotional’ dimensions of social structures, so ‘racial life’ is ‘suffused with shared passions, imageries and fantasies’ that inform modes of ‘attachment, defence, solidarity or struggle’ within society. These emotional responses guide political action at the structural level, but also individual, group and institutional actions, as practices are laden with racialised meaning and associated emotional content. All of the above provide a contextual framework for social action through the production and enactment of particular social, economic, political, symbolic and emotional positions.

If structural racism is seen as a way of accounting for the more abstract workings of culture, economy and society, a micro‐sociological focus on interpersonal racism is attuned to the more routine, everyday expressions of racism, which prey upon and accentuate marginal racialised statuses (Essed 1991, Knowles 2003). Rather than being seen to exist independently of structures, it is through interpersonal actions that the social‐structural, cultural and collective‐emotional aspects of ‘racial orders’ are actualised – albeit they are also framed by these wider contexts (Emirbayer and Desmond 2015). As Knowles (2003) argues, ‘people are the motor of race making’ as ‘racial orders are in fact composed of myriad and ordinary everyday social processes and mechanisms with which people interface’. Similarly, forms of interpersonal racism operate within collectives, such as families, neighbourhoods or institutions, providing them with a structural character (Phillips 2010). In this sense, structural racism operates through the interpersonal, not outside of it; structural racism may shape the terrains of everyday racialised and racist interactions, but is itself also an outcome of cumulative patterns of everyday racism (Essed 1991). Consequently, there exist interdependencies between structural and interpersonal racism.

Understanding race/ethnic inequalities also requires attention to be paid to the role of institutional racism. First coined by Carmichael and Hamilton (1967) ‘institutional racism’ was used to highlight how racialised inequalities were not naturally occurring, but a function of actions operating within institutions. Institutions have a particularly important role, located as they are at the meso‐level between the structural and the interpersonal. Institutional settings represent sites where we see the concentration and mediation of structural forms of disadvantage and interpersonal racism (Bailey *et al*. 2017, Emirbayer and Desmond 2015, Phillips 2010). Conceptually, ‘institutional racism’ has been beset by the challenge of attributing racism to institutions, rather than to individuals (Bradby 2010). However, by locating ‘institutional racism’ within a wider nexus involving structural and interpersonal processes, we can see how institutional practices are produced both via ‘agential overt and unwitting practices of individuals’ and ‘interacting causal structural conditions’ (Phillips 2010). Indeed, the idea that ‘institutional racism’ is really a problem of ‘interpersonal racism’ ignores the ways in which ‘institutional and interpersonal racism interpenetrate and support one another’ (Emirbayer and Desmond 2015), even if they are analytically distinct. Recognising this interpenetration and support, allows us to avoid the detachment of institutional practices from the actions of individuals ‘as if it concerned qualitatively different racism rather than different positions and relations through which racism operates’ (Essed 1991). So, we can consider how the systems of operation in institutions relate to and reproduce both structural and interpersonal racism, and how this is reflected in routine activities, situated knowledge and the collective‐emotional structuring of relationships and institutional cultures, resulting in discriminatory policies and practices that impact on both staff and users of services. And, of course, race/ethnic inequalities in staffing may impact directly and indirectly on inequalities in the provision of services.

## The impact of interpersonal racism on risk of severe mental illness

It is clear that interpersonal experiences of racism and discrimination are present in the lives of race/ethnic minority people in the Global North. Given the diverse and often very subtle forms that interpersonal racism takes, it is extremely difficult to quantify the level of risk faced by ethnic minority people (Karlsen and Nazroo 2006). In addition, quantified assessments typically focus on individual experiences at a single point in time, so fail to capture how experiences of racism and discrimination operate across, and impact on, the life courses of connected individuals. Nevertheless, such assessments do show high levels of risk within the UK, levels that have not changed meaningfully over the past 20 years. For example, 15 per cent of Black Caribbean people reported experiencing racist abuse, assault, or vandalism in 1993/1994, compared with 14 per cent in 2000 and 12 per cent in 2008/2009 (Karlsen and Nazroo 2014, Virdee 1997). Similarly, 20 per cent of Black Caribbean people were very, or fairly, worried about being a victim of a racist attack in both 1993/1994 and 2008/2009 (Karlsen and Nazroo 2014, Virdee 1997). Indeed, qualitative studies that examine the significance and meaning of racism clearly indicate how central such experiences are to the lives of ethnic minority people (Stevens *et al*. 2012, Virdee 1995, 1997). Underlying these experiences is a worrying continuation of prejudice in the majority population within the UK, which has remained at a consistent and high level over the past 30 years (Kelley *et al*. 2017).

It is also important to recognise that interpersonal incidents of racism are an attack on communities, rather than just individuals (Virdee 1997). Racism need not have been experienced personally for it to produce a sense of threat (Karlsen and Nazroo 2004). As Oakley (1996) points out: ‘the distinguishing feature of racial violence and harassment is not simply that it involves members of different racial groups or ethnic groups; it is that the action is racially motivated… . Racially motivated behavior, therefore, is not an attack aimed at a person purely as an individual, but an attack on a member of a category or group’. Indeed, acts of racism are reflections of historical legacies of racial orders and domination, so their psychological impacts are to reinforce the disempowerment and lack of security of racialised identities.

It is clear that the threat associated with these events, whether or not directly experienced, impacts on the health of race/ethnic minority people. This has been documented in several countries across the Global North, as evidenced in recent reviews (Paradies 2006, Paradies *et al*. 2015), as well as within the UK (Karlsen and Nazroo 2002a, 2002b, 2004, Wallace *et al*. 2016). In the context of severe mental illness, this relationship has been shown also for the risk of psychosis. For example, in one study using data from the mid‐1990s, Karlsen and Nazroo (2002b) show that those reporting to have experienced racist verbal abuse had a prevalence of psychosis almost three times that of people reporting no harassment, while this prevalence was almost five times higher for those reporting to have experienced racist physical abuse. Similarly those who believed that the majority of British employers would discriminate against someone on the grounds of race/ethnicity had a prevalence of psychosis that was more than 50 per cent higher than those who did not. These findings were echoed in a later study, where Karlsen *et al*. (2005) show that risk of psychosis was doubled for those who reported an experience of racist verbal abuse or physical assault.

## Structural inequalities and risk of severe mental illness

Processes related to racism and discrimination result, both directly and indirectly, in inequalities in accessing economic, physical and social resources and consequent inequalities across a range of related outcomes. Within the UK there are deep and persisting ethnic inequalities across almost all socioeconomic dimensions – income, employment, residential location, housing and education (Jivraj and Simpson 2015, Modood *et al*. 1997), which have recently been thoroughly documented in the UK Government's Cabinet Office led Race Disparity Audit (for up‐to‐date data see: http://www.ethnicity-facts-figures.service.gov.uk). For example, an examination of Census data over the periods 1991, 2001 and 2011 shows that Black Caribbean and Black African men and women have had persistently high levels of unemployment over this 20‐year period, more than twice as high as the White rate (Kapadia *et al*. 2015). And while Pakistani and Bangladeshi men and women have seen large falls in unemployment over the period 1991 to 2011, they continue to have much higher unemployment rates than White men and women, and any fall is mainly a result of a large rise in part‐time work (Kapadia *et al*. 2015). For Bangladeshi men the part‐time employment rate has risen from just over 3 per cent in 1991 to 35 per cent in 2011, a figure that is coupled with a fall, rather than a rise, in full‐time employment rates and that is seven times higher than that for White men (Kapadia *et al*. 2015).

It might seem reasonable to expect such inequalities to have diminished over time, particularly for populations that have a history of migration, such as ethnic minority groups in the UK. For example, a second generation would be more fluent in English and would have passed through the UK education system. Also, the introduction of equality legislation, which has been in place in the UK for more than 50 years, might be expected to have diminished the negative outcomes of discrimination. There is some suggestion that this is the case in relation to education, where the improvements in educational attainment that occurred in the UK for all ethnic groups over the period 1991 to 2011 were smallest for the White group, leading to a narrowing of ethnic inequalities. For example, the proportion of White people with a degree‐level qualification increased from 13 per cent in 1991 to 26 per cent in 2011, while that for Indian people increased from 15 per cent to 42 per cent and that for Black Caribbean people increased from 9 to 26 per cent (Lymperopoulou and Parameshwaran 2015). Indeed, in 2011 people from most ethnic minority groups were more likely than White British people to have degree‐level qualifications and less likely to have no qualifications (Lymperopoulou and Parameshwaran 2015).

However, such an improvement is not the case across the board (Jivraj and Simpson 2015), and the data on employment, described above, indicate that these relative improvements in educational attainment for ethnic minority people have not translated into equivalent improvements in employment outcomes. This emphasises the depth and persistence of structural inequalities in relation to race/ethnicity and the difficulties in changing relevant processes. Improvements in some outcomes (in this case educational attainment) do not necessarily translate into improvements elsewhere (in this case employment, but also housing and probability of living in a deprived area) (Jivraj and Simpson 2015), despite the implementation of a range of legislative and equal opportunities processes.

Of importance here is that the resulting levels of economic, social and geographical inequality make substantial contributions to ethnic inequalities in health outcomes (Nazroo 1998), including severe mental illnesses (Nazroo 1997, Nazroo and King 2002). Qassem *et al*. (2015), for example, clearly identify the social and economic disadvantages faced by Black people to be at the root of their higher risk of psychotic illness. Although they do not put it in these terms, they point to the ways in which racialised Black identities increase risk of economic hardship, unemployment, discrimination and harassment. To this we could add the significance of living in deprived neighbourhoods and in poor quality housing, the accumulation of these disadvantages and insecurities across a life course and the impact of this ongoing disadvantage on one's identity.

## Explaining the gap – the consequences of institutional racism?

Almost all of the research undertaken in this field points to the greater risk of psychotic illnesses among ethnic minority people in the UK (and elsewhere), and this is particularly the case for Black people. However, a very puzzling finding is the large difference between the estimate of the increased risk of severe mental illness derived from clinical studies of incidence compared with estimates from surveys examining prevalence in community settings. How to make sense of the difference in these estimates is not immediately obvious. Typically, the problem is addressed through a focus on methodology, an examination of how robust the findings are when using these two approaches.

Any attempt to estimate the prevalence, or incidence, of rare and difficult to identify conditions in a group that makes up only a small minority of the population raises obvious difficulties. Over more than 20 years, methods for obtaining reliable and valid estimates of the prevalence of psychosis within a defined population have been developed (Meltzer *et al*. (1994) provide the first application of these methods). These rely on the use of screening instruments, validation of diagnoses for a subset of those screened positive using structured clinical interviews, and then using these data to estimate prevalence (Nazroo 1997). Similarly, methods have been developed to obtain good probability samples of ethnic minority populations, even if their relative numbers in the population are small (Modood *et al*. 1997). As far as can be determined, these approaches provide reliable and valid estimates for the defined population, but they have two important drawbacks. First, they are population estimates that at an individual level contain a large degree of uncertainty – the majority of those who screen positive do not in fact reach what might be called clinical criteria for a diagnosis. So, prevalence is estimated using probabilistic methods rather than case finding. This means significant measurement error at an individual level, so the estimate of the causal role of individual risk factors also contains considerable uncertainty, even if those factors are measured well. Second, the population under study is likely to be defined in ways that exclude some of those most at risk of psychotic illness, such as those in prisons, those who are in psychiatric institutions and those who do not respond to surveys. If there are differential rates of risk in institutionalisation, or survey non‐response, across populations being compared, then the comparisons will contain meaningful error. And, of course, ethnic minority people are much more likely to be present in prisons and psychiatric institutions and are, on average, more likely to not respond to surveys.

Similarly, estimates using clinical incidence studies have become increasingly robust. Initial concerns about biases in diagnostic practice and in case note reviews have been addressed by using reviewers who are blinded to demographic characteristics and who ascertain caseness using standardised criteria. Concerns about underestimates of population denominators have been addressed by the careful use of population census data that, since 1991 in the UK, have included ethnicity. And, although not extensive, what evidence there is suggests that diagnostic categories are valid across ethnic, cultural and language groups (Heuvelman *et al*. 2018, Nazroo 1997). Of course, problems remain. Such studies carry the assumption that all incident cases of psychotic illness will come to the attention of health services within a short period and that none of the cases that are identified as incident are in fact recurrent. Neither assumption may hold (Bresnahan *et al*. 2007), and, again, the sources of bias in incident case identification might vary across ethnic groups. In addition, making case notes blind to demographic characteristics is not straightforward – race/ethnicity may be written into case notes in ways that are impossible to extinguish.

Rather than focusing on methodological issues, we suggest that it is fruitful to consider what these rates are estimates of when comparing them. For community surveys this is clear. They are estimates of prevalence of the outcome under consideration (in this case psychotic illness) in the community resident population. In the case of incident studies, though, the nature of the research means that they are not studies of incidence per se, but studies of incidence resulting in certain forms of treatment – typically admission to a hospital. So, the estimated incidence rates combine both incidence of illness and pathways to care. We might then conclude that the additional increase in estimated relative risk for incident diagnosis over prevalence studies reflects differences in pathways to care – that a Black person with an incident psychotic illness is more likely, maybe as much as three times more likely, to be admitted to a public psychiatric institution for treatment. This, we might argue, reflects the processes of institutional racism within relevant systems – criminal justice, social work and health care – which then results in a greater likelihood for a Black person compared with a White person to be admitted into a psychiatric institution and consequently receive a diagnosis of a psychotic illness.

## The operation of racism within institutions – pathways through care

The outcomes of institutional racism can be seen in the greater likelihood of race/ethnic minority people to have more negative pathways through care, poorer access to effective interventions, and poorer outcomes. There is evidence of all three of these in relation to severe mental illness. A recent systematic review and meta‐analysis of studies from England revealed that Black Caribbean patients are almost three and a half times more likely than White patients to experience compulsory admission under the powers of the Mental Health Act, with a rate that is just over three times higher for Black African patients and one and half times higher for South Asian patients (Halvorsrud *et al*. 2018). Similarly, Black Caribbean patients are more than two and a half times more likely, and Black African patients more than three and half times more likely, than White patients to have contact with the police prior to admission (Halvorsrud *et al*. 2018). And Black Caribbean patients are almost three times more likely, and Black African patients almost twice as likely, than White patients to have involvement in the criminal justice system (Halvorsrud *et al*. 2018). Given this, it is not surprising that Black patients are more likely to be in psychiatric intensive care units and medium secure units, and more likely to be secluded or physically restrained (SCMH 2006). In contrast, both Black Caribbean and Black African patients are much less likely, almost half as likely, to have contact with a general practitioner prior to admission compared with White patients (Halvorsrud *et al*. 2018).

Although the evidence on this has been consistent over several decades, investigations into why this might be so have been limited in number and focus. One clear and perhaps surprising finding, given the intended protective nature of the powers under the Mental Health Act, is that the excess detention is despite evidence that prior to admission Black Caribbean patients are less likely than White patients to display evidence of self‐harm and are no more likely to be aggressive to others (Harrison *et al*. 1989, McKenzie *et al*. 1995, Rogers 1990). Indeed, psychiatrists seem more likely than police to consider Black Caribbean patients who have been detained in an emergency as dangerous to others (Rogers 1990). There is also some evidence that once admitted Black Caribbean people are more likely to be perceived by staff as potentially dangerous (Rogers 1990), perhaps as a result of fear inducing stereotypes such as that of ‘Big, Black and dangerous’ (Keating 2007).

Despite this negative, compulsory, route into treatment, there is the possibility that treatment received is equitable and effective. However, although the Mental Health Act includes provision for treatment using psychological and social interventions, the very ethos of such treatments becomes undermined by the coercion involved and consequent loss of trust, meaning that the main treatment approach will, by necessity, be medication within a narrow model of care. Indeed, evidence suggests that Black Caribbean patients with psychosis are less likely than White patients to receive psychologically based interventions or antidepressants (Das‐Munshi *et al*. 2018, McKenzie *et al*. 1995). In addition, Black patients are just over 50 per cent more likely to be prescribed with injectable antipsychotic drugs than White patients, and among those with treatment resistance Black patients were almost half as likely to receive the recommended medication (clozapine) than White patients (Das‐Munshi *et al*. 2018). Perhaps not surprising then, are the findings that Black Caribbean patients with a diagnosis of psychosis remain in acute hospital care longer than White patients, have more frequent compulsory readmissions and have more frequent outpatient follow‐up contacts, despite having fewer negative symptoms (Keating 2007, Takei *et al*. 1998). In addition, Black patients are over‐represented in assertive outreach services that have the power to impose supervised treatment orders on patients who do not engage with treatment in community settings (Patel *et al*. 2011). The coercive nature of these services undermines personal agency and autonomy for patients living in the community.

These negative treatment pathways might reflect the difficulty of providing services in the deprived contexts where many race/ethnic minority patients live, rather than being the result of institutional racism. It is clear that such areas have less resourced, more disorganised and poorer quality service provision (Weich *et al*. 2012), increased levels of in‐patient treatment (Keown *et al*. 2018) and an increased risk of detention under the Mental Health Act (Weich *et al*. 2017). However, the ability of those who are commissioning and providing services to tolerate such circumstances requires them to distance themselves from those receiving services. This is easier in the context of service provision to members of a group, or an area, that is racialised – the ‘othering’ of such groups and places enables the necessary distance to be achieved. This ‘othering’ is also the condition that gives rise to and helps to sustain inequalities in the operation of institutions. Our experience of working in this field has shown that this allows unequal outcomes to be understood as a consequence of general structural conditions within the context of resource constraints, so creating a powerful set of conditions where race/ethnic inequalities are considered the norm, beyond the control of commissioners and practitioners, and, consequently, more easily accepted.

Returning to the risk of detention under the Mental Health Act, we argue that while structural conditions of socioeconomic disadvantage and racism create an increased risk of severe mental illness, these conditions also shape encounters with institutions that have policies and practices that lead to unequal outcomes across race/ethnic groups. The consequent inequalities should be considered to be a result of interacting and interdependent structural, interpersonal and institutional racisms. Indeed an integrated understanding of the operation of racism at macro, meso and micro levels provides a more comprehensive identification of how racisms operate to shape opportunities, and a powerful and fundamental framework for understanding race/ethnic inequalities more generally. We suggest that the neglect of such a thorough assessment of the role of racism has led to the failure of the development and implementation of policy in this area, as evidenced by the outcomes of the Inside Outside report (Sashidharan 2003), the Delivering Race Equality Action Plan (Department of Health 2005) and the recommendations from the Joint Commissioning Panel for Mental Health (2014).

## Concluding comments

In the context of inequalities in risk of severe mental illness, Qassem *et al*. (2015) argue for a greater investment in resources for mental health services for areas with a higher proportion of Black Caribbean and Black African people. In this they implicitly follow the arguments of Singh and Burns (2006), who assert that the greatly higher risk of admission to hospital with a diagnosis of psychosis for Black people reflects differences in risk of illness, although Qassem *et al*. (2015) additionally suggest that the discrepancy may additionally result from the greater needs of Black people with psychosis. Singh and Burns (2006) go somewhat further, claiming that: ‘Construing racism as the main explanation for the excess of detentions (under the Mental Health Act) among ethnic minorities adds little to the debate and prevents the search for the real causes of these difference’. They also argue that coercive treatment should not be seen as punitive, rather ‘The Mental Health Act is an enabling act: it allows services to ensure that treatment is available for those most in need of it’ (Singh and Burns 2006). Although their conclusions come from different lines of argument, in essence both Qassem *et al*. (2015) and Singh and Burns (2006) are, in effect, arguing that current inequalities can be addressed by greater investment in mental health services.

Rather than going along with this line of argument, we contend that an integrated approach to understanding how racism shapes the increased risks of experiencing severe mental illness for race/ethnic minority people and their more adverse pathways through care allows for a more fundamental understanding of causal processes, one that goes beyond a singular focus on individual experiences of discrimination, or institutional practices, and instead situates an enquiry within a wider analysis of racism, racialisation and inequality. There are two important policy conclusions to draw from our review of relevant evidence, that was conducted within this novel theoretical framework. First, that Black Caribbean and Black African people do face an increased risk of psychotic illness and an increased risk that is driven by racially based social and economic disadvantage, reflecting both structural and interpersonal racism. Second, that the significant discrepancy between that increased risk and the much higher risk for Black Caribbean and Black African people of hospital admission and treatment for a psychotic illness reflects institutional racism. Indeed, it is worth noting Fernando's (2004) comment that ‘It is in the field of forensic psychiatry that racial injustices and cultural oppression are most acutely felt by black and Asian service users’. Here Fernando points to the complex, coercive and adverse pathways into, through and out of care faced by race/ethnic minority patients, and he goes on to conclude that: ‘the main and perhaps most serious problem is institutional racism that pervades all major systems affecting British people, including mental health services and the main disciplines that inform such services, namely psychology and psychiatry’ (Fernando 2004). We do not argue that institutional racism is somehow distinct from structural and interpersonal racism, rather we suggest that the systems of operation in institutions are shaped by (and reproduce) structural and interpersonal racism. Institutions are sites crucially situated in and shaped by both wider forms of structural racism and inequality, and spaces within which forms of interpersonal racism and micro‐forms of racialisation operate, and can sediment and acquire greater salience precisely through their institutionalisation. This becomes reflected in routine activities, situated knowledge and the collective‐emotional structuring of relationships and institutional cultures, which then shape discriminatory policies and practices, and the actions of individuals, resulting in inequalities in the experience of racialised groups.

It might be argued that the analysis we offer is relevant only to the racialised context of UK mental health services. Rather, it seems likely that the processes that we identify here operate in other contexts, given the similarities in the ways that ethnic minority, and particularly Black, people are racialised in the Global North, and similarities in the higher risk of hospital admission for severe mental illness in those countries where it has been studied. It also might be argued that these processes are specific to the way that mental health institutions address severe mental illness. Here it is worth considering how institutional racism might operate differently across institutions with a different focus – the functions of institutions dealing with cancer screening, for example, are likely to result in very different forms of institutional racism, perhaps organised around notions of individualised responsibility for risk management in the context of fiscal constraint. This suggests that the novel, integrated, theoretical approach to the analysis of the impact of racism on race/ethnic inequalities in health that we offer in this article could be usefully applied to other health outcomes, and could also be applied to race/ethnic inequalities more generally. For example, it is likely that the processes we identify here are relevant to other institutions concerned with surveillance, control and the management of risk to others, such as criminal justice, social work and education. Indeed, it is worth considering how the practices of such institutions sit alongside and reinforce each other.

This, then, suggests a research agenda focussed on the ways in which particular, and inter‐related, institutions produce and reproduce racial/ethnic orders and consequent inequalities. This requires a focus both on how such inequalities operate within institutional structures, for example in employment practices, and on how institutional racism shapes the provision of services and the experiences of clients. It also requires a focus on the contexts and functions of institutions, so how an institution relates to broader social structures and operates in particular contexts, and how different institutions relate to each other – how the boundaries between institutions operate. Such a research agenda has the potential to be focussed on policy, to operate in partnership with both the clients and the leadership of institutions, with a view to consider how the operation of institutions can be reformed to address race/ethnic inequalities, rather than to reproduce them.

In conclusion, we have argued that racisms are fundamental causes of observed race/ethnic inequalities in risk of severe mental illness and in outcomes relating to severe mental illness. In order to account for these inequalities, it is important to examine the ways in which structural, interpersonal and institutional racisms operate and mutually constitute one another. We can see how racisms operate upon and through racialised identities, with the actions of individuals and institutions being both shaped by and informing wider structural forms of racism. Here, racism is not the sole preserve of any one of these domains. Indeed, ideas of a separation between ‘racial structure’ and ‘racial agency’ (in interpersonal and institutionalised form) are ‘best replaced by an outlook that regards those elements as reciprocally constituting moments of a unified social process’ (Emirbayer and Desmond 2015). Given this, alongside a focus on other sources of social and economic inequality, it is crucial that the public health agenda pays close attention to issues of racism and how they shape the lives of race/ethnic minority people. Indeed, we suggest that public health should adopt an anti‐racism agenda and, in this case, place this centrally in discussions around the reform of mental health systems.

## Funding

Lankelly Chase Foundation.
